# Emerging Adjuvants for Cancer Immunotherapy

**DOI:** 10.3389/fchem.2020.00601

**Published:** 2020-07-30

**Authors:** Hong-Guo Hu, Yan-Mei Li

**Affiliations:** ^1^Key Lab of Bioorganic Phosphorus Chemistry and Chemical Biology, Department of Chemistry, Tsinghua University, Beijing, China; ^2^Beijing Institute for Brain Disorders, Beijing, China; ^3^Center for Synthetic and Systems Biology, Tsinghua University, Beijing, China

**Keywords:** adjuvant, immunotherapy, immune activation, pattern recognition receptors, cancer

## Abstract

Cancer is a life-threatening disease, and immunotherapies have been developed as a novel, potent treatment for cancer. Adjuvants, used alone or in combination with other agents, play crucial roles in immune activation. This is necessary for cancer immunotherapy, particularly in the construction of therapeutic cancer vaccines. Adjuvants activate antigen-presenting cells and promote the presentation of antigen epitopes on major histocompatibility complex molecules, further enhancing adaptive immune responses, including cytotoxic T lymphocytes, to elicit cancer-cell death. However, the applications of adjuvants are limited by their poor efficacy or insufficient safety. In recent studies, researchers attempted to develop safe, efficacious adjuvants for cancer immunotherapy, and many compounds (including inorganic compounds, organic molecules, polymers, and colloids) have been identified and optimized as agonists of various pathways. In this review, we focus on the discovery and structural design of emerging adjuvants and discuss how these findings benefit healthcare.

## Introduction

During infection, the immune system is activated by pathogens harboring pathogen-associated molecular patterns (PAMPs), including agonists of toll-like receptors (TLRs), NOD-like receptors (NLRs), and stimulator of interferon (IFN) genes (STING). PAMPs can be used as adjuvants in the treatment of infections and other diseases because of their potential to enhance the immune response by combining with pattern recognition receptors (PPRs) on antigen-presenting cells (APCs) (Petrovsky and Aguilar, 2004; Wang et al., 2013). Immunotherapy is a promising method for the treatment of various diseases, including infections, autoimmune diseases, and cancers, and adjuvants are powerful agents for immunotherapy (Reed et al., 2013). In recent years, many novel types of adjuvants have been identified and applied for the development of cancer vaccines (Cai et al., 2013; Shao et al., 2018; Wu et al., 2018). However, for further clinical use, newly emerging adjuvants need to overcome various challenges, including evaluation of pharmacodynamics and their adverse reactions.

In this review, we summarize newly discovered adjuvants, including inorganic nanoparticles, organic molecules, and polymers. For consideration of more widely recognized and applied adjuvants, please refer to relevant references (Broz and Monack, 2013; Li et al., 2018; Wu et al., 2019).

## Emerging Adjuvants

### Inorganic Compound-Based Adjuvants

#### Inorganic Nanoparticle-Based Adjuvants

In the last two decades, multifunctional inorganic nanomaterials have become a major focus in the biomedical field owing to their applications in imaging, tumor therapy, and drug delivery (Chen et al., 2019; Eppler and Jewell, 2019; Meel et al., 2019). Some inorganic nanomaterials act as immunostimulants by promoting the activation and maturation of immune cells (Sun and Xia, 2016; Li et al., 2018). The composition, size, morphology, and charge of these nanomaterials can affect their adjuvant activity. Accordingly, inorganic nanoadjuvants are usually within a certain size range and have a specific shape.

Pathogens induce immune responses by triggering a “danger sensing” signal via PAMPs (Aderem and Ulevitch, 2000). However, the physical cues of the microbes that trigger the immune system are still unclear. Wang et al. (2018b) showed that TiO_2_ nanoparticles decorated with nanospikes activated innate immunity by physical stimulation. During their phagocytosis by APCs, mechanical stress induced by the spiky particles disrupted the cell membrane resulting in K^+^ efflux, which activated the inflammasome and subsequently stimulated innate immunity. When combined with ovalbumin (OVA) protein, spiky TiO_2_ nanoparticles functioned as a potent adjuvant and promoted adaptive immunity for cancer immunotherapy (Wang et al., 2018b).

Ferumoxytol, a United States Food and Drug Administration (FDA)-approved iron supplement, inhibited mammary tumor growth and lung cancer metastases. Ferumoxytol-treated macrophages displayed a higher level of pro-inflammation Th1-type mRNA and M1 macrophage polarization. After incubation with ferumoxytol and macrophages, adenocarcinoma cells showed higher caspase-3 activity for tumor cell killing (Zanganeh et al., 2016). Gu et al. (2019) have studied the mechanism of macrophage activation by iron oxide and found that magnetite induced macrophage activation and M1 polarization to a greater extent than hematite via activation of the IFN regulatory factor 5 rather than inducing nitric oxide synthase through the nuclear factor-κB pathway (Gu et al., 2019). Trujillo-Alonso et al. (2019) found that ferumoxytol could be used to treat a mouse model of leukemia that was associated with low ferroportin levels. The lack of ferroportin in leukemia cells resulted in ferumoxytol accumulation, and the high iron levels disrupted the balance between oxidant production and scavenging. As a result, reactive oxygen species were increased, inducing death of leukemia cells and improving survival (Trujillo-Alonso et al., 2019).

#### Manganese Salt Adjuvant

Immune cells use PPRs to defend against infections by triggering innate immune responses. STING is an important PPR for anti-infection responses and immunoregulation. Agonists of STING include cyclic dinucleotides (CDNs; e.g., CDA and CDG) and their mimics. Additionally, Jiang's group reported manganese as a regulator for the STING pathway (Wang et al., 2018a). As one of the most abundant metals in mammal tissues, manganese is involved in many physiological processes, including antioxidant defense, neuronal function, and immune responses (Horning et al., 2015; Kwakye et al., 2015), by regulating Mn-dependent enzymes. Mn^2+^ alerts immune cells to infections by increasing the sensitivity of cyclic GMP-AMP synthase (cGAS) and STING. When infections occur, Mn^2+^ released from various organelles and accumulated in cytosol. The accumulated Mn^2+^ then enhances enzymatic activity of cGAS and sensitivity to double-stranded DNA by binding to cGAS. The liberated Mn^2+^ also enhances the activity of the STING pathway by improving the binding affinity of cGAS-STING. Additionally, Mn^2+^ is a potent immune stimulator, and in the absence of any infection, it induces type I IFN responses and promotes cytokine production (Wang et al., 2018a). Thus, Mn^2+^ is a potential adjuvant for immune activation.

Based on these findings, a jelly-like manganese colloid (MnJ) adjuvant was developed as a potent adjuvant for humoral, mucosal, and cellular immune responses without obvious side effects (Zhang et al., 2019). In many antigen models, MnJ promoted antigen presentation and enhanced cytotoxic T lymphocyte responses. Pathway studies revealed that the adjuvant activity of MnJ was related to both the NLRP3 inflammasome and STING activation. In a mouse model, the production of OVA-specific antibodies and activation of CD8^+^ T cells were enhanced in MnJ-OVA-treated mice, indicating that MnJ was a potential adjuvant for cancer immunotherapy.

Nanomaterials are mainly exogenous, and detecting these molecules can act as a danger signal for the innate immune system. Based on their properties of immune stimulation, many nanomaterial-based adjuvants have emerged for cancer immunotherapy. Another superiority of nanomaterials is their tissue targeting (Wilhelm et al., 2016), including enhanced permeability and retention in tumors (Fang et al., 2011). However, nanomaterials *per se* may cause some side effects. For example, TiO_2_ nanoparticles can promote breast cancer cell metastases by inducing endothelial leakiness (Peng et al., 2019).

There are some meaningful details that remain unclear about the journey of nanomaterials through the body, and exploring the uptake, migration, and clearance of such materials in the microenvironment, or in different cells, tissues, and organs, would be conducive to clinical research. The MnJ adjuvant holds great potential for clinical use because of its ability to activate STING without distinct side effects in mice. Nevertheless, substantial additional work is needed before this treatment can be used on humans.

### Organic Molecule-Based Adjuvants

The immune system plays important roles in preventing pathogen infections. PPRs on immune cells recognize PAMPs from pathogens then boost the immune responses for pathogen clearance. These PPRs include TLRs, NLRs, RIG-1-like receptors, STING, and C-type lectin receptors (Broz and Monack, 2013). Many adjuvants are PAMPs with definite structures, and these molecules induce immune activation by interacting with PPRs (Akira et al., 2001; Wang et al., 2013). Emerging small molecule-based adjuvants include modified PAMPs, new ligands for PPRs, and agents of new pathways.

#### Agonists of TLRs

TLRs are type I transmembrane proteins that regulate the innate and adaptive immune responses. There are 10 functional TLRs in humans (12 in mice), and these TLRs have various agonists (Wang et al., 2013). Exploring these agonists and their derivatives as adjuvants has contributed to the development of cancer immunotherapy (Tom et al., 2019).

**Figure 1:**
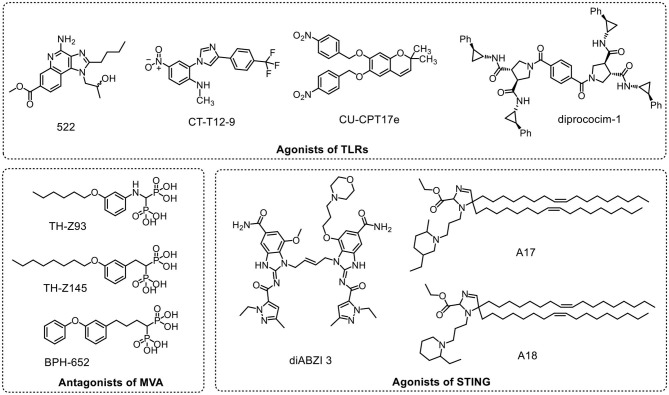
Structures of emerging adjuvants.

Structure–activity relationship (SAR) analyses of TLR7/8 and the FDA-approved agonist imiquimod demonstrated that N1-, C2-, and C7- were important for the activity of imiquimod. After comparing the immunostimulation of imidazoquinolines with different modifications at N1-, C2-, and C7-, a novel TLR7/8 agonist (522, Figure 1) was found to induce high levels of pro-inflammatory cytokines (Schiaffo et al., 2014). 522 was applied to cancer immunotherapy following encapsulation in polymeric nanoparticles (Kim et al., 2018). High-throughput screening is a simple, rapid method for active molecule identification and drug discovery. After screening of a 24,000-compound library using an interleukin (IL)-8 luciferase reporter cell line expressing human TLR2 receptors whose ligands are lipopeptides (such as Pam_3_CSK_4_ and Pam_2_CSK_4_), five compounds were chosen as candidates for TLR2 agonists (Guan et al., 2010). Based on these candidates, the Yin lab showed that *N*-methyl-*4*-nitro-2-[4-(4-nitrophenyl)1*H*-imidazol-1-yl] aniline (GA) interacted with TLR1/2 rather than TLR2/6. To achieve high selectivity and efficacy, GA was optimized using SAR studies to obtain a novel compound, CU-T12-9 (Figure 1). CU-T12-9 showed a higher affinity for TLR1/2 and potent TLR1/2 signaling pathway activation (Cheng et al., 2015). By measuring tumor necrosis factor (TNF)-α released from THP-1 cells, diprovocims were discovered from a ~10,0000-compound library. After comprehensive SAR studies, the most potent agonist, diprovocim-1 (Figure 1), was identified. Diprovocim-1 acted as a TLR1/2 heterodimerization promoter and enhanced the immune responses through the TLR1/2 signaling pathway (Morin et al., 2018). In a mouse model, diprovocim-1 plus OVA immunization significantly promoted antigen cross-presentation and evoked cellular immune responses. Through synergistic interactions with anti-PD-L1, the diprovocim-1 adjuvant efficiently eliminated melanoma in mice (Wang et al., 2018c). From the Maybridge HitFinder v11 library, Zhang et al. (2017) identified a small molecule, 17e (Figure 1), as an agonist for multiple TLRs. 17e synergistically activated TLR3/8/9 on human TLRs expressed in HEK 293 cells, and 17e inhibited the growth of HeLa and HuMEC cells (Zhang et al., 2017). Overactivation of the immune system induces systemic inflammation and results in inflammatory diseases (Taniguchi and Karin, 2018). To reduce systemic inflammation during immune activation, Li and colleagues designed a photoswitchable Pam_3_CSK_4_ derivative (P10) and showed that it could regulate inflammation and immune activation by optical control of the heterodimerization of TLR1/2 (Hu et al., 2019).

#### Antagonists of the Mevalonate (MVA) Pathway

The MVA pathway is an important metabolic pathway (Goldstein and Brown, 1990) that is responsible for hypercholesterolemia (Dimmitt et al., 2017) and bone disorders (Russell, 2011). Clinical research showed that interruption of the MVA pathway with statin and bisphosphonate drugs evoked immune responses (Drenth et al., 1999), suggesting that targeting the MVA pathway may be druggable for fimmunotherapy. In mouse and cynomolgus monkey models, Xia et al. (2018) showed that lipophilic statin drugs and rationally designed bisphosphonate molecules (TH-Z93, TH-Z145, and BPH-652; Figure 1), which interact with three different enzymes (farnesyl pyrophosphate, geranylgeranyl diphosphate synthase, and squalene synthase) in the MVA pathway, acted as efficient adjuvants. In contrast to conventional adjuvants that trigger “danger sensing,” these inhibitory adjuvants blocked the geranylgeranylation of Rab5 in APCs, resulting in prolonged antigen preservation. Thus, the secretion of IFN-γ by both CD4^+^ T cells and CD8^+^ T cells was increased for tumor killing. In tumor models, these inhibitory adjuvants resulted in better treatment outcomes than CpG (a TLR9 agonist) with or without anti-PD-1 (Xia et al., 2018).

#### Agonists of STING

STING plays vital roles in innate immunity (Burdette and Vance, 2013) and is a promising target for cancer immunotherapy (Wu et al., 2019). After viral infection, cGAS recognizes cytosolic dsDNA and then produces cGAMP for STING activation. Activated STING induces the production of type I IFN and other pro-inflammatory cytokines, which are necessary for innate immune responses and adaptive immunity (Galluzzi et al., 2018). However, CDNs (such as CDA and CDG), the native ligands of STING, show poor stability and transmembrane permeability, thereby limiting their applications (Wu et al., 2019). Current studies of STING agonists are aimed at modifying CDNs and their intratumoral delivery; these works have been well summarized (Su et al., 2019; Wu et al., 2019). However, non-nucleic acid agonists of STING have not been reported frequently. A set of small molecules (ABZIs) that bind to the C-terminal domain of STING have been identified through high-throughput screening of competition with cGAMP (Ramanjulu et al., 2018). After structural optimization, these researchers generated diABZI 3 (Figure 1) that exhibited enhanced STING binding energy and activation ability. Intravenous injection of diABZI 3 inhibited tumor growth significantly and improved survival in a mouse model of colorectal cancer, indicating that the non-nucleotide diABZI 3 could have potential applications in cancer immunotherapy.

Anderson's group has focused on the development of mRNA vaccines for cancer immunotherapy (Kauffman et al., 2016). During the optimization of delivery materials, Miao et al. (2019) obtained adjuvant-like lipids from over 1,000 lipid formulations; these lipids were similar in unsaturated lipid tails, cyclic amine head groups, and dihydroimidazole linkers. The top candidate formulation triggered a strong immune response, which further confirmed that these cyclic lipids activated immune cells through the STING pathway. Vaccines containing promising lipid adjuvants (A17 and A18; Figure 1) boosted the maturation of APCs and triggered T-cell responses for tumor immunotherapy (Miao et al., 2019).

#### Synergistic Agonists

Synergistic activation may represent a new paradigm for adjuvant and vaccine design (Xu and Moyle, 2018; Tom et al., 2019). In recent years, Kahn's group focused on exploring covalently linked TLR agonists, such as dimeric (covalently coupled TLR2 and TLR9 heterodimers) (Mancini et al., 2014) and trimeric agonists (TLR4, TLR7, and TLR9 covalently linked agonists) (Tom et al., 2015) agonists. Zom et al. (2019) synthesized a peptide conjugate that could simultaneously trigger the NOD2 and TLR2 signaling pathways and enhance the production of TNF-α, IFN-γ, and IL-2 by murine T cells (Zom et al., 2019). Exploration of the different TLR combinations required for the synthesis of various di- or tri-agonists is the major challenge. To solve this problem, Albin et al. (2019) developed a panel for synthesizing tri-agonists through a set of bioconjugation reactions. The core of the panel was a triazine modified with an alkyne, an amine, and either a maleimide or carboxylic acid, which could be linked to TLR agonists containing azide, carboxyl, thiol, or amino groups. Five tri-agonists were constructed according to the panel to explore the potential of TLR combinations (Albin et al., 2019). They found that the linker length between the two agonists affected immune activity, and the influence of the linker length depended on the properties of the agonist (Ryu et al., 2016). Multi-adjuvant formulations improved immune activation and enhanced antitumor efficacy. Li's group also developed a self-assembly method to construct nano-immunostimulants by taking advantage of the electrostatic interactions between Pam_3_CSK_4_ and CpG. Assembling nano-immunostimulants with antigens yielded multicomponent vaccines, which induced robust antitumor responses in a mouse model (Sun et al., 2016, 2017).

The defined chemical structure and mechanism of organic molecule-based adjuvants endow each type of adjuvant with special functions. The diversity of small-molecule adjuvants makes each one optional for different diseases. Furthermore, combining various adjuvants leads to the development of multifunctional and powerful agents. However, except for their efficacy, safety is the major criterion of new agents. Nonspecific small-molecule-based adjuvants would cause undesired inflammation, and targeting ligands or delivery systems would be helpful to improve both the efficacy and safety these organic adjuvants.

### Polymer-Based Adjuvants

Polymer materials are widely used in biological tissue engineering, imaging, and drug delivery (Sun and Xia, 2016; Rodell et al., 2018; Shae et al., 2019). However, few studies have reported the intrinsic immunostimulation properties of these polymers. The Gao lab has studied nanomaterials and nanoarchitectures for the diagnosis and targeted treatment of cancers. Based the “proton sponge” concept, they developed a library of polymers that were ultra-pH-sensitive (UPS) over a broad pH range (4.0–7.4) (Ma et al., 2014). The UPS polymer nanoparticle, ONM-100, was licensed to OncoNano Medicine and a phase 1 clinical trial of ONM-100 as an imaging agent during surgery is in press. The optimized UPS polymer nanoparticle, PC7A, triggered a strong cytotoxic T-cell response for cancer killing. Investigation of the mechanism demonstrated that the PC7A nanoparticle activated STING and enhanced immune responses, which was related to the cyclic seven-membered ring on the side chain. Moreover, the nanovaccine (PC7A loaded with peptide antigens) enhanced the efficiency of cytosolic delivery and antigen cross-presentation. Thus, the STING-activating nanovaccine enhanced cancer immunotherapy by boosting antigen-specific CD8^+^ T cells (Luo et al., 2017, 2019).

Polymer nanoparticles that play important roles in drug delivery and have the ability of immune activation would make polymers more powerful tools in cancer immunotherapy. However, considering both efficacy and safety, polymers for clinical use must meet FDA standards. Before introducing polymers for cancer treatment, a complete evaluation of their pharmacokinetic and biodistribution profiles is necessary.

## Discussion

The immune system is critical for mammalian defenses against infections and diseases. Thus, exploring mechanisms of immunoregulation and exploiting immunoregulators to benefit cancer treatment are important research areas. However, progress in the discovery of vaccine adjuvants has been slow, and few adjuvants have been approved by the FDA for medical applications. Before moving forward to clinical research, more attention should be given to the pharmacokinetics and safety of these agents, especially to determine the uptake, metabolism, and adverse reactions associated with each candidate agent. Another issue is how to optimize the formulation and administration of adjuvants and antigens. Other than simply mixing, optimizing ways of delivery and injection may strengthen the immune activation and antitumor effects of adjuvants. Utilizing adjuvants to enhance cancer immunotherapy is meaningful, but the most important thing is to develop safe and effective adjuvants, a process that requires substantial basic research.

## Author Contributions

All authors listed have made a substantial, direct and intellectual contribution to the work, and approved it for publication.

## Conflict of Interest

The authors declare that the research was conducted in the absence of any commercial or financial relationships that could be construed as a potential conflict of interest.
